# Gender and postcolonial studies: history of the concept and debate

**DOI:** 10.3389/fsoc.2024.1414033

**Published:** 2024-07-10

**Authors:** Irene Strazzeri

**Affiliations:** Department of Human and Social Sciences, University of Salento, Lecce, Italy

**Keywords:** sexuality, ecofeminism, decoloniality, gender, post colonials

## Abstract

The debate on the concept of gender in postcolonial studies is extremely complex and involves a variety of theoretical and practical perspectives. Postcolonial studies has shown the connection between gender identity, colonial power, and decolonisation processes. This paper will explore the social construction of gender in colonial contexts, the way in which colonial practises have influenced gender dynamics, and the struggles for resistance and freedom in which women and gender-nonconforming people have engaged in postcolonial countries. The issue will be raised of how gender is interpreted and experienced in different cultures and social contexts. Furthermore, the analysis of colonisation and decolonisation processes will provide a starting point to understand how gender hierarchies have been built and criticised in postcolonial contexts, leading to the development of the most recent ecofeminist and decolonial perspectives.

## 1 Introduction

Gender studies is an interdisciplinary approach to the study of the sociocultural meanings of sexuality and gender identity. Developed in North America between the 1970s and the 1980s in the field of cultural studies, it spread in Western Europe in the 1980s. Gender studies originated in a branch of feminism and was influenced by post-structuralism and French deconstruction, by research on the relationship between psychology and language. A significant role was also played by gay studies and postmodernism. Gender studies is not a field of knowledge *per se*, but a mode of interpretation. It is the result of a combination of different methodologies that embrace diverse aspects of human life, the construction of identity, and the relationship between the individual and society, the individual and culture. For this reason, a gender-sensitive interpretation focused on gender aspects can be applied to any field of human and social sciences.

Since its early days, gender studies has been characterised by political emancipation aspects. It is strongly connected with the condition of women and other subaltern subjects. Far from just proposing theories and using them to analyse culture, gender studies aims to introduce some changes in the dominant mentality and society. It is connected with the movements for the emancipation of women, lesbian and gay people, ethnic and linguistic minorities. It deals with issues linked to racial and ethnic oppression, the development of postcolonial societies, and globalisation.

Individuals are traditionally divided into men and women based on their biological differences, with sex and gender being often considered the same thing. Conversely, gender studies suggests a theoretical distinction between these two aspects. Sex refers to physical and anatomical biological traits that lead to the differentiation between males and females, whereas gender, as a cultural construction, is a representation that goes beyond the biological makeup, creating the status of man or woman. Sex and gender are not conflicting, but rather interdependent dimensions. Therefore, biological traits become the basis of a process of construction of gender identities, which are the result of the persistent social and cultural strengthening of identities: through a series of interactions that tend to define differences, a symbolic boundary is constantly drawn between men and women. At a social level, one’s gender needs to be continuously proven, through one’s behaviour, language, and social role, which has led to the development of the concept of gender roles. Gender is a learned, rather than an innate, trait. However, as postcolonial studies has suggested, the relationship between sex and gender changes based on geographical areas, historical periods, and cultures, with masculinity and femininity being dynamic concepts that need to be historicised and contextualised.

## 2 The sex/gender system

Each society decides what values should be attributed to different gender identities, thus defining the concepts of man and woman, which are hence relative. In her *The Traffic in Women*, published in 1975, anthropologist Rubin (1975) describes such a phenomenon by coining the phrase ‘sex/gender system’, a system through which biological data are transformed into social fate. This is an asymmetrical binary system in which the masculine holds a privileged position compared to the feminine, to which it is strongly interconnected, with the system providing a reciprocal definition of them both. Gayle Rubin’s studies spread throughout Europe, leading to diverse outcomes.

In the mid-1970s, the characteristics of the concept of gender identified two types of it. The term was originally considered binary, in a flexible rather than univocal sense. It does not describe the female condition, but the social construction of one’s sex. The female condition cannot be analysed without taking into account the male one. Therefore, gender translates into a concept that implies reciprocity and dialectical relationships. This changed the interpretive frameworks used by social scientists and paved the way for unprecedented levels of investigation and interpretation. The concept of gender extended to economic analysis and the division of labour, not in sectoral terms, but as a redefinition of the workforce. Gender is not an additional concept, as it redefines and critically re-examines a whole. If the analysis of the changes occurring in the institutions, reproduction systems, and cultural dimensions of society takes into account that they evolve and organise in a sexed way, then perception increases. According to Amartya Sen, the importance of gender as a parameter is crucial in socioeconomic analysis and it is complementary to, rather than competitive with, class, ownership, occupation, income, status, and ethnicity variables (Sen, 1993, p. 73). The transforming component of the concept seems to be evident, as it does not imply a neutral perspective on sexed reality, but the acknowledgement that the latter is characterised by imbalance (Mohanty, 2015).

As Scott (1996, p. 42) has pointed out, gender is the dimension where power reveals itself. The difference between the sexes is socially and historically constructed as disparity in the workplace, in the intellectual and symbolic sphere. Evidence of such an imbalance can be found in the gender discrimination fought against by feminist movements, which have challenged male supremacy. The development of a new paradigm has therefore transformed into an essential tool for an analysis of inequalities that focuses on the socially constructed aspects of sexual inequality and the non-biological factors contributing to gender disparity. The emphasis on gender has resulted in a new concept of relationality and a combined idea of the feminine and the masculine. However, two critical aspects should be highlighted. On the one hand, the concept represents the crystallisation of feminist thought in Western culture. On the other hand, the concept has become an object of debate and change that is influenced by criticism, self-criticism, practical aspects, and reflection.

## 3 Gender: from the concept to an analytical perspective

The replacement of the concept of sex with that of gender has left numerous problems unsolved, since the changes in the gender experience seem to be difficult to analyse, with the identification of the subject becoming increasingly problematic. This is also connected with the fact that the deconstructivist approach has not been embraced in all the fields and groups in which feminist thought developed. The philosophy of sexual difference, which developed in Italy and France in the 1970s, holds that sex remains the main aspect to define the self (Irigaray, 1990). Nevertheless, the concept of gender is never static and immobile. Nor men or women suffer their fate without reacting. Therefore, the differences that gender encompasses correspond to dynamic phenomena that the subjects continuously transform by acting and reflecting on themselves.

One may hence wonder what perspective the sexes provide in the historical process and in relation to change. To answer this question, both essentialism and deconstruction should be taken into account. According to Derrida, the only process responsible for the existence of two genders is the historico-social construction, interpreted as a continuous accumulation and stratification of symbols and meanings (Derrida, 1967). Western logocentrism has resulted in objects, rather than subjects, that remain stuck in the cultural practises related to the context they belong to. Therefore, gender as a social construction may be deconstructed and freed from itself: women can get rid of the discourse that has traditionally described them, showing its misleading nature. On the other hand, the debate on existentialism is connected with that on the crisis of the contemporary subject. Whilst Kristeva (1973) has argued that women are safe in their non-identity, Michel Foucault has maintained that scepticism should be shown towards the construction of any collective (female) subject that is able to act and actively participate in the transformation of social aspects (Foucault, 1969). However, as it has been pointed out by John Scott, whose theories have been embraced by numerous social scientists, discrimination is never just the result of symbolic action. It concerns institutions, economic relationships, and the distribution of power (Scott, 1996).

Regardless of the different analytical perspectives, the theoretical starting point seems to be both historically and culturally rooted in Western philosophy, where male thought has imposed itself. Considered universal and neutral, it defines the world starting from itself. This has deprived the feminine of both its access to the symbolic and its ability of self-signification. Such a reconstruction has occurred through the acknowledgement of ‘the irreducible’, the fact that it is fundamental for women to be sexed in a context of difference. As Cavarero has highlighted, the body is both the physical and symbolic origin of the woman (Cavarero, 1987, p.180). The female being can only affirm herself by challenging the monopoly of knowledge held by men, which makes her development asymmetrical. The evolution of the female subject gradually began to be characterised by a plurality of differences, in line with postmodernism of Lyotard (1979).

In response to postmodernism, Benhabib, Fraser et al. (2017) have suggested the reconstruction of the subject, introducing a more precise—albeit variable—concept of gender. Being aware of the past and the constraints of the present, they have explored the similarities between the concept of gender and that of difference in the postmodern framework. They have tried to reconnect the biological and the social: far from being an entity, the body is an experience, which means that the meaning of the sexes may be shaped and changed. No longer seen as the basis on which identity is built, one’s biological makeup starts to be considered a variable. The identification of the limitations of the binary character imposed on the sexed human subject has opened up a gap in the falsely neutral male thought, breaking its seeming unity (Rich, 1977). Going beyond a dichotomous perspective means fostering the process of female identification, promoting freedom and change. The production of difference both in the self and society is the distinctive trait of modern history (Walzer, 1984, p. 37). In other words, another analytical perspective developed. However, further clarifications and a significant mediation approach are needed in order to better understand the theoretical-political shift described. Indeed, no theory can be said to be unitary and valid for all women, no elite can represent universality. Experience and knowledge can take multiple forms, which always need to be situated and contextualised.

## 4 Unresolved issues: women’s and postcolonial studies

A gap has been opened up also in Western feminism, due to the claims made by ethnically different, sexed subjectivities. The female reflection on the successful anti-colonial and nationalist struggles has questioned the unilateral perspective of the Western feminist movement and experience (Strazzeri, 2021). Similarly, in the context of the North American feminist movement, female intellectuals of colour and the lesbian movement have distanced themselves from Western social elites (Davis, 1981). This has resulted in a kind of nomadic feminism that has travelled the world, crossing borders. Women belonging to the most diverse cultures have shared ideas and interests, whilst highlighting differences. Simultaneously, a multiculturally-oriented sociological and political perspective has developed (Taylor, 1994). As a result of an analytical effort, postmodern feminism has embraced a perspective that is similar to the one that sociology has long adopted. Social complexity has been acknowledged, as well as the existence of multiple subjects, the universal nature of values, and other aspects that, being already available, could not be ignored, not even as a result of a strange division of tasks. Within this epistemological framework, the subtle shift in focus that has occurred in the concepts of gender, subject, woman, and man may be re-examined. These have started to be considered immanent and transient meanings that are the result of the processing of experience, in the awareness that context can change. Women have contributed to a change that mirrors their identity. Therefore, gender is both the result and the starting point of a process of social construction that acts in order to transform the conditions it identifies. Two important issues revolve around gender: self-definition, which is a cognitive action, and self-projection, a political act.

The gender perspective has also been embraced in other fields of sociological discourse, including women’s and men’s studies, which do not focus on women and men, respectively, but rather explore their relationship at a macro and micro level. Also men’s studies has rejected the idea of defining a biological entity. In famous study on the transformation of intimacy of Giddens (1995), the analysis of the modern democratic culture is completed through the reclaiming and exploration of the emotional sphere of both genders: as anatomy stops being destiny, sexual identity becomes a way of life, as long as there is an equal exchange between quality of life, time management, social stratification, and flexibility.

Since the 1980s, feminism has embraced some new perspectives. The last decades have been characterised by the debate on the difference between feminism, in the singular, and feminisms, in the plural. Such a debate is interconnected with another great change, which occurred when women’s thought entered the academic field, with the development of Women’s and Postcolonial Studies. Women’s studies has extended the concept of gender, highlighting the social construction of genders and their relationship: far from being biological data, sexed genders are the result of human history, they can be continuously re-thought and re-built. As Rich (1977) would put it, heterosexuality is not compulsory.

The theoretical process that has led to the defeat of gender has been guided by Judith Butler, who has proposed a performative theory that is based on the Freudian idea that personal identity is shaped by the concept of normality: the thesis is that gender performativity forms gender. According to Butler, the construction of the sexed, desiring subject is not a choice, but the result of the regulative discourse. Without a deconstruction of sex, the strategy based on the sex/gender system is ineffective in combating discrimination (Butler, 2004).

Power determines the understanding of the female gender also within the feminist movement. Black Feminism has denounced the racism and eurocentrism characterising Western white feminism, which sees the rest of the world as a periphery. Pointing out how the material experience of African American women has been cancelled, Angela Davis has simultaneously criticised racism and sexism by combining narrative, theoretical, and autobiographical aspects (Davis, 1981).

In the 1990s, feminism developed in the South of the world, in countries such as India, South Africa, Latin America, and the Middle East. It acknowledged the differences between women and denounced the imposition of a single model of liberation and emancipation.

Following the end of European colonialism, a decolonisation process started with Derrida’s (1967), Said’s (1991) Fanon’s (2015)and works. The analysis of colonialism helps to understand the present through the acknowledgement of the power relationships between the Western and the Third World in the context of the global hegemony of the Western culture. The Western world considers Third World women a single, monolithic subject that is sexually subordinate, ignorant, poor, influenced by family and religious traditions, subdued and victimised. The other variable in the relationship is the Western woman, described as educated, modern, and free. Subaltern women seem to be stuck between the nationalist interests of indigenous patriarchy and those of colonial governments.

## 5 Perspectives for a decolonial ecofeminism

The methods of postcolonial and anti-imperialism feminist criticism have resulted in reflections focused on the connection between the different struggles for freedom taking place in the world, beyond national borders, in an attempt to lead to a rethinking of feminist practises of transnational solidarity. According to Chandra Talpade Mohanty, these are the main issues dealt with by postcolonial feminism, or at least by a feminism based on anti-racism and anti-capitalism at a transnational level (Mohanty, 2003). This can be described as a feminist process because it aims at building alliances and fostering solidarity through gender, race, and social class, whilst trying to identify the national and sexual strategies that resulted in the subjugation of women in colonised countries. Mohanty belongs to the post-independence generation, a generation that is aware of colonial (institutional) spaces and constraints, but also of the practises of *decolonial* movements, of the global and national struggles for freedom, and especially of the motivation generated by the postcolonial perspective, as though it were a generational mandate.

For my post-independence generation, such a mandate consisted in seeing theoretical problems as decolonising issues, through the development of a strong awareness: consciously decolonising spaces and analysing the impact of such a practise. For instance, revising the official high school syllabus, which only included the history of the British Kingdom and disregarded our local history, the origins of our community, led me to explore the tools of power, what makes it visible, natural, and normative (Mohanty, 2015, p. 164).^1^

As Mohanty’s words show, decolonisation is not a formal process that only consists in overthrowing governments to replace them with local elites. It is a more revolutionary process that transforms the structures of the self, the community, and the government at any level. It implies a rethinking of the practises of resistance in order to counteract the social and psychological domination of imperialism. Far from being an individual process, this is a collective initiative characterised by shared reflections and questions to answer.

One of the most significant contributions to postcolonial feminist theory has been made by Gayatri Chakravorty Spivak, a Bangladeshi philosopher who has raised awareness of the conditions of Third World women. Spivak’s objective is the development of a new, ‘postcolonial’ feminism that may distance itself from the pre-existing structures. The use of the adjective ‘postcolonial’ helps to focus on the South of the world, making colonised and exploited women the subject of a new feminism. In other words, postcolonial thought is a radical form of thematization of the needs of women coming from poor countries. In order to clarify the conditions in which women live in developing countries, in her *A Critique of Postcolonial Reason*, Spivak (1999) tells the storey of two women who were close to her.

The first storey tells about the Rani of Sirmur. Little is known about her life, which Spivak explains by stating that: ‘[t]his, then, is why the Rani surfaces briefly, as an individual, in the archives; because she is a king’s wife and a weaker vessel, on the chessboard of the Great Game’ (Spivak, 1999, p. 231). One may wonder who this Rani actually is. It should be clarified that ‘Rani’ is the Hindu term for ‘queen’, or more precisely, the word refers to someone whose role can be said to be similar, albeit not identical, to that of a Western queen. In 1820, near the Himalayan hills, in the region of Sirmur, a Rani lived, the wife of a Rajah that had been deposed by the British due to his debauchery and bad behaviour. Widowed, the young woman was established as the immediate guardian of her underage son. According to Hindu rituals, a widow had the possibility—the duty—to perform *sati*, by sacrificing herself on her husband’s funeral pyre. ‘For the female ‘subject’, a sanctioned self-immolation within Hindu patriarchal discourse, even as it takes away the effect of ‘fall’ attached to an unsanctioned suicide, brings praise for the act of choice on another register’ (Spivak, 1999, p. 235). In other words, a Hindu widow was pushed by her family to commit suicide, thus promoting the patriarchal system in which she lived. That is why Spivak unsurprisingly states that ‘[a]s we approach Sirmur, we move from the discourses of class and race into gender’ (Spivak, 1999, p. 231). However, the Rani is not the only woman in the Hindu socio-political organisation that can be considered an example of the subaltern Third World woman. Spivak also recalls what happened to a distant relative of hers, Bhubaneswari Bhaduri. The storey is once more set in India, but in 1926. The young woman hanged herself in her father’s flat in Calcutta, for no apparent reason. About 10 years later, a letter she had written to her sister revealed that she had been commissioned to carry out the assassination of a politician, a task she had failed to accomplish. What Spivak highlights is that the girl decided to commit suicide whilst she was menstruating, in order to prevent her family from mistaking her last cry for help for the act of a girl that had inappropriately got herself pregnant (Spivak, 1999, p. 307).

By analysing the storeys of these women that were ignored by history and silenced by their culture, Spivak realises that these are symbols of social and cultural domination, social and sexual hierarchies, resistance and deception (Spivak, 1999, p. 317). Spivak describes Third World women, exemplified by the two ones mentioned, as ‘subaltern’ (Spivak, 1999). The image of the subject, or better, of the subaltern object, is that of indigenous Hindus that, ruled and controlled by the British, had been deprived of a space where they could express their own identity. Women were faced with an even worse situation, as they experienced a double subaltern condition: on the one hand, like men, they belonged to a culture—the Hindu one—that had been colonised and exploited; on the other hand, due to their sex, they were subjugated by men, marginalised, and prevented from voicing their needs. Such a reflection has led Spivak to focus on the attempts made by Western feminists to emancipate the women living in the South of the world. Western feminism should mainly be blamed for having failed to counteract the dynamics of power and domination of imperialism and capitalism, thus becoming complicit in the exploitation of colonised countries. As Chandra Talpade Mohanty (2003) has pointed out, evidence of such an attitude can be found in the stereotypical image of Third World women presented by Western feminists. Feminists living on the wealthy side of the planet have created an incomplete and humiliating image of their poorer sisters, failing to investigate the actual conditions and needs of Third World women. Western feminism has described subaltern women as being sexually subordinate, ignorant, influenced by family and religious traditions, and lacking critical thinking skills. Therefore, postcolonial feminism is characterised by a relativity of positions that fail to consider the multiplicity of female experiences. Spivak has attempted to emphasise the misleading idea that feminism is an easily classifiable movement, in order to encourage thinkers to identify, through mutual respect and the understanding of difference, the power relationships underlying the global cultural and socioeconomic order that prevents the total emancipation of both Western and subaltern women.

When it comes to postcolonial feminism, such a difficulty is mainly due to the heterogeneous nature—in terms of time and space—of the intellectual contributions and political efforts that are at the basis of the variety of feminist studies labelled as ‘postcolonial’. A geographical difference should be highlighted. Although without embracing the vehement criticism that some scholars have made of the academic institutionalisation of postcolonial studies—as it has been in the case of Terry scathing review of Eagleton (1999) of Spivak’s work—it should be pointed out that most of what is labelled as postcolonial feminism is the result of analyses conducted in English and American academic institutions by scholars who left the Third World. In other words, the traditional narrative of such a movement does not include any analysis carried out outside conventional intellectual circles. However, this spatial and symbolic distribution mirrors a more general feature of contemporary postcolonial geography, as the traditional distinction between centre and periphery—First and Third World—can no longer grasp the complex relationship between the local and the global that has resulted from the globalisation of both capital and migration. Postcolonial female writers strongly defend their being at the margins of the centre, or similarly, their being at the centre of the periphery they describe and from which they express their views (Puwar, 2003).

Such geographical issues are complemented by matters related to a series of works that have entered the international feminist debate in a confrontational way (Hooks, 1981; Mohanty, 1988). From the opposite sides of the English-speaking Atlantic world, two dimensions have emerged that are linked to the colonisation of discourse by white, middle-class feminists, to the detriment of their counterparts. The first of such dimensions is the absolutisation of the difference between emancipated women, seen as masters of their own destiny, and passive victims, who benefit from, or are the object of, Western intervention. The second dimension is the paradoxical cancellation of difference in light of a hypothetical ‘universal sisterhood’.

Such bitterness may only be overcome over time, with a stricter *politics of location*. Yet, these bitter feelings are not just the result of the contrast between different intellectual purposes, but also the outcome of a radical difference in the fight against, and resistance to, patriarchal domination. They have increased throughout history, whenever the inseparability of race and class, nature and culture, has been emphasised in the subjective experience of patriarchal oppression. Despite their different experience of colonialism in the strict sense of the term, African American feminists have made an undeniable contribution to the development of postcolonial feminism. Numerous of them have pointed out how white women were complicit in the establishment of slavery, highlighting the ambiguity with which the first American feminist organisations engaged in the fight for its abolition (Davis, 1981). Postcolonial feminist criticism has denounced the complicity of mainstream Western feminism, and British feminism in particular, in legitimising colonialism as a mission aimed at civilisation and expropriation of resources. A significant part of such reflections explores the impact that policies of modernisation have had on colonised populations, with measures aimed at replacing ‘traditional’ forms of social organisation, production systems, sexual division of labour, and social reproduction. They have revealed how the monoculture of crops has paralleled the monoculture of the mind, intended to sacrifice biodiversity (Spivak, 2010). The complex concepts of race, gender, and sexuality in the colonial context have also been analysed, as well as their retroactive effect on the social stratification of the mother country. Part of the reflection on the role of gender subordination in colonial domination, and vice versa, focuses on the possibility of giving voice to subaltern women, who have been silenced by colonial and even nationalist and anti-colonial narratives. This explains why, whilst investigating *sati*, Spivak asked herself that question that would become the title of her most famous work—*Can the Subaltern Speak*? Both her question and the way she answered that sparked off an intense debate and prompted further criticism. ‘There is no space from which the sexed subaltern subject can speak’ (Spivak, 1988, p. 307).

Another common thread in postcolonial feminism is the idea of writing as a place of expressing, or better developing, a hybrid and unstable identity that is the opposite of the dominant phallogocentric one, a concept developed by French feminism thanks to Cixous and Irigaray (Ives, 2018). The necessary, and always necessarily incomplete and unsatisfactory, translation of the self into the language of the Other becomes the way to experiment with new possibilities, new freedoms, and even new forms of consciousness: this is the meaning of the *conciencia de la mestiza* (Anzaldua, 1987), a new sensitivity and awareness. The sharp contrast between identity and experience implies, through the effects of an increasingly radical deconstruction, a different idea of feminist political identities and the possibility of a fight for the protection of the common good. The politics of identity becomes more and more the result of what Spivak calls ‘strategic essentialism’, that is, the result of strategic emphasis placed on the attributes of an identity that cannot be reduced to the single factors of its subordination—social class, race, and gender. The sisterhood lie is replaced by a promise of solidarity and contingent alliances that still need to be built (Mohanty, 2003), in an attempt to ethically construct a global identity.

One of the main issues that militant ecofeminists are trying to resolve is how to translate that into both a new transnational feminist movement that may protect common good and different interests, as Mohanty hopes, and a productive way to politically deal with global and local differences. The macro-narrative aimed at destabilising the well-established constructions of knowledge and power created by Western colonial tradition shows their inner flaws, the voices they have ignored and silenced, their contradictions, their unexpected breaking and crisis points. The inappropriate female Other (or Self) constantly oscillates between stressing her being similar to other women, in her being different, and reminding herself of her being different, destroying any well-established definition of organic alterity that may be in line with an ecopolitics of change. In this controversial and ambiguous space that is the expression of disorientation, division, and pain, but also of new life opportunities and unexpected critical scenarios, the challenge still is to understand how the emancipation of critical thought from ethnocentrism may deconstruct the Western model of development in the perspective of subalternity.

## Author contributions

IS: Conceptualization, Writing – original draft, Writing – review & editing.

## Footnotes

^1^Our translation.
